# Rippl and the Alzheimer’s Association: Enhancing Community Support for Caregivers and People Living with Dementia

**DOI:** 10.1002/alz.095812

**Published:** 2025-01-09

**Authors:** Katie Evans

**Affiliations:** ^1^ Alzheimer’s Association, Chicago, IL USA

## Abstract

N/A N/A N/A N/A N/A N/A N/A N/A

